# Medial Temporal Lobe Roles in Human Path Integration

**DOI:** 10.1371/journal.pone.0096583

**Published:** 2014-05-06

**Authors:** Naohide Yamamoto, John W. Philbeck, Adam J. Woods, Daniel A. Gajewski, Joeanna C. Arthur, Samuel J. Potolicchio, Lucien Levy, Anthony J. Caputy

**Affiliations:** 1 Department of Psychology, Cleveland State University, Cleveland, Ohio, United States of America; 2 Department of Psychology, George Washington University, Washington, District of Columbia, United States of America; 3 School of Psychology, University of Wollongong, Wollongong, New South Wales, Australia; 4 Department of Aging and Geriatric Research, Cognitive Aging and Memory Clinical Translational Research Program, Institute on Aging, University of Florida, Gainesville, Florida, United States of America; 5 Office of Basic & Applied Research, National Geospatial-Intelligence Agency, Springfield, Virginia, United States of America; 6 Department of Neurology, George Washington University Medical Center, Washington, District of Columbia, United States of America; 7 Department of Radiology, George Washington University Medical Center, Washington, District of Columbia, United States of America; 8 Department of Neurological Surgery, George Washington University Medical Center, Washington, District of Columbia, United States of America; University of Münster, Germany

## Abstract

Path integration is a process in which observers derive their location by integrating self-motion signals along their locomotion trajectory. Although the medial temporal lobe (MTL) is thought to take part in path integration, the scope of its role for path integration remains unclear. To address this issue, we administered a variety of tasks involving path integration and other related processes to a group of neurosurgical patients whose MTL was unilaterally resected as therapy for epilepsy. These patients were unimpaired relative to neurologically intact controls in many tasks that required integration of various kinds of sensory self-motion information. However, the same patients (especially those who had lesions in the right hemisphere) walked farther than the controls when attempting to walk without vision to a previewed target. Importantly, this task was unique in our test battery in that it allowed participants to form a mental representation of the target location and anticipate their upcoming walking trajectory before they began moving. Thus, these results put forth a new idea that the role of MTL structures for human path integration may stem from their participation in predicting the consequences of one's locomotor actions. The strengths of this new theoretical viewpoint are discussed.

## Introduction

An important function of vision is to facilitate navigation. As important as this function is, however, visual information is frequently degraded or made unavailable by occlusions or poor lighting conditions. This being the case, it is advantageous for sighted individuals to remain able to navigate without vision, and humans certainly have this ability. For example, the average human can sight a target up to 20 m away or more, and then walk to it accurately in an open field while blindfolded–although responses tend to become more variable as the target distance increases, observers typically reach the target with very little systematic error [1], [2]. This kind of non-visual navigation precludes using visible landmarks to determine one's position, so the brain must rely upon internally generated (idiothetic) self-motion information, such as vestibular and proprioceptive signals. This process is known as path integration or dead reckoning [3], [4]. Good performance in the blindfolded walking task indicates that the brain is finely tuned to sense and integrate the on-going self-motion signals when walking along linear trajectories.

As we will show, there is much evidence that brain structures in the medial temporal lobe (MTL) participate in key cognitive functions associated with path integration (e.g., spatial representation, self-motion sensing, and temporal processing). However, the scope of the MTL's role in path integration remains unclear. To what extent is the MTL involved in human path integration? More specifically, to what extent does the MTL participate in *on-line* path integration–that is, updating relative to locations that must be remembered for only a few seconds (as opposed to well-learned landmarks stored in long-term memory)? The experiment reported here addressed these issues by testing various kinds of self-motion sensing and integration in patients who have undergone unilateral resection of the MTL as therapy for epilepsy. If the MTL plays a crucial role in these components of path integration, therapeutic resection of these structures should result in observable deficits in path integration.

### Key components of path integration

#### Spatial representation

Effective path integration entails forming a representation of the current displacement from one's last known position. There is abundant evidence that the MTL plays an important role in representing spatial information in memory [5]–[11], especially when retention intervals are longer than several seconds. Significantly, however, it has also been shown that the MTL is critical for creating a spatial representation of the surroundings even within the temporal range of short-term memory ([12]–[16], but see also [17], [18]). This suggests that the MTL makes contribution to on-line path integration beyond its well-established role in longer-term storage of spatial information.

#### Self-motion sensing

Some neurons in the MTL dynamically change their firing rates depending on an individual's location or heading within an environment, even when vision is occluded (i.e., place, grid, and head direction cells) [19]–[29]. This suggests that the MTL's role in navigation includes sensing and tracking self-motion on the basis of idiothetic signals. Consistent with this view, MTL-injured humans exhibit navigation deficits [30]–[33], and functional neuroimaging studies show activation in the hippocampus or parahippocampal gyrus during navigation in virtual environments ([34]–[36]; see also [37]). Many of these studies involve more than just self-motion sensing–for example, they draw upon relatively long-term spatial memory and visual scene recognition. Thus, even though a role for the human MTL in navigation and path integration has been widely discussed, the role of the MTL in self-motion processing per se remains poorly understood. Significantly, however, path integration based on optic flow has been shown to engage the hippocampus, among other regions outside the MTL [38], [39].

#### Temporal processing

To determine one's position on the basis of idiothetic self-motion information, one must integrate these signals over time. Hippocampal lesions disrupt memory for duration [40], [41] and temporal order [42]–[45]. Patients with circumscribed bilateral hippocampal damage show impairments both in memory for durations after delays of 4–20 s and in estimation of durations of 8–20 s [46]. Although deficits in time perception might impact on-line path integration, this linkage has not been explored.

### MTL role during on-line path integration

Despite the clear participation of MTL structures in the aforementioned components of human path integration, their role in path integration on relatively short time-scales is unclear. Evidence of impairments in MTL-injured patients during short-duration path integration tasks has been inconsistent. Wiest et al. [47] and Worsley et al. [48] found deficits in some tasks involving body rotations (e.g., route reproduction) but not others (e.g., turn reproduction). Philbeck et al. [49] found evidence of linear path integration deficits, whereas Worsley et al. did not. Kim et al. [50] and Shrager et al. [51], meanwhile, found no path integration deficits. Thus, a critical link between the MTL and path integration remains in doubt.

One possible reason for these apparently inconsistent findings is that the scope of the MTL's role for path integration may be more narrowly focused than has been proposed. If this were the case, MTL damage would not cause across-the-board impairments in any tasks involving path integration; instead, it should result in selective impairments in particular tasks. This view is potentially important for accommodating the disparate findings because path integration tasks that have been studied in humans differ in their component processes. For example, a typical path integration task involves previewing a target and subsequently walking to it without vision (target-directed walking) [1], [49], [52]. This task allows participants to create a spatial representation of the target location, predict the walking path, and engage in active control of locomotion. By contrast, in another common path integration task, blindfolded participants are guided by an experimenter and estimate distance and direction of their locomotion (experimenter-guided walking) [48], [49], [52]. In this task, the lack of foreknowledge about the destination makes it impossible for participants to predict and select their upcoming trajectory, removing an essential element needed for active control of locomotion. Such a difference in task demands might modulate the degree of engagement of the MTL during path integration, given its role in predicting locomotion paths that are taken in the immediate future ([53]; see also [54]–[58]): in these studies, place cells in the rodent hippocampus that are tuned to fire at locations along the future locomotion trajectory showed increased activity *before* the animal started traversing the trajectory. In humans, it has been shown that path integration is facilitated when neurologically intact individuals are able to anticipate their upcoming trajectory and control their locomotion more actively [59]–[62], suggesting that trajectory prediction does exert observable effects on human path integration. Furthermore, as discussed previously, there is evidence that the MTL plays a role in forming spatial representations of the surroundings [12]–[16]–representations that are presumably necessary for predicting a locomotion trajectory. All of these previous findings converge on the possibility that MTL damage would have a greater adverse effect on the target-directed walking task than on the experimenter-guided walking task.

Path integration tasks also differ in the sensory modalities that they involve. Some tasks, such as target-directed walking and experimenter-guided walking, encompass many kinds of self-motion signals, including arm and leg proprioception and vestibular signals from otolith organs. In other tasks, path integration is performed on the basis of a single source of information such as optic flow [38], [39], [63] and vestibular signals from semicircular canals [47]. It is possible that the role of the MTL for on-line path integration is quite general, in that it integrates a variety of sensory signals over time to support estimation of body displacement. If this is true, MTL-injured patients should exhibit deficits in tasks that require integrating self-motion signals, regardless of which sensory modality provides the signals. Alternatively, MTL structures may be less important for integrating some types of self-motion than others, a possibility that predicts modality-specific effects of MTL damage. At least in some cases, the MTL appears to play a role in processing purely visual or vestibular self-motion signals [26], [38], [39], [47], but the range of sensory modalities integrated by the MTL remains poorly understood. Modality-specific effects of MTL damage could arise from multiple causes (e.g., differences in frames of reference used for encoding spatial information, rather than differences in sensory inputs per se), but nevertheless, testing diverse tasks of path integration that differ in sensory modality stands to provide crucial basic data for future investigations targeting these topics.

### Rationale for the present study

Importantly, consistent with the view that the MTL contributes to path integration via its role in predicting future locomotion paths, our previous work showed that patients with the right MTL lesions were impaired at the target-directed walking task but not at the experimenter-guided walking task [49]. Specifically, the patients walked significantly farther than neurologically intact age-matched control participants when attempting to walk to a previewed target in target-directed walking, whereas these two groups did not differ in their estimates of walked distance in experimenter-guided walking. However, because only these two path integration tasks were tested, the previous data would not allow us to draw a clear conclusion as to whether trajectory prediction is the primary MTL function through which it participates in human path integration. It is possible that factors other than prospective processing of upcoming trajectories (e.g., modalities of self-motion signals, as discussed above) are of greater importance in determining the MTL's involvement in path integration. In addition, given the novelty of the idea that human MTL structures are engaged in path integration to the extent that tasks allow individuals to anticipate their upcoming walking paths, it is imperative to replicate the critical findings (i.e., impaired target-directed walking and intact experimenter-guided walking) and ensure their reliability before we make any attempts to interpret them.

To address these issues, we administered a variety of behavioral tasks, including target-directed walking and experimenter-guided walking tasks, in epilepsy patients who had undergone therapeutic unilateral resection of MTL structures. These individuals were compared to neurologically intact age-matched control participants. We focused on their performance in the tasks at short durations (e.g., 8 s or less). This is the temporal range at which some studies have found relatively little impact of MTL lesions [50], [51] and others have demonstrated systematic deficits [47]–[49].

### Overview of tasks

Following our past work [49], [52], we used four primary tasks in the study described below. Visual perception and spatial memory were assessed independently of path integration by obtaining (a) verbal distance estimates and (b) delayed distance matches of visible targets. Path integration was assessed independently of visual perception and memory by (c) guiding blindfolded participants along a straight path and then asking them to estimate the distance traveled (experimenter-guided walking). Performance in these tasks was compared with (d) walking without vision to a previewed target (target-directed walking), a task that provides a separate assessment of visual perception, memory, and path integration. By giving a preview of the target, this task allows observers to predict the extent of their upcoming self-motion and compare their incoming self-motion signals during locomotion with this prediction. Being able to make this comparison may reduce the effect of limitations or variability in the incoming sensory information and therefore can improve estimates of the current location of the body [64], [65].

To explore the possible role of the MTL in integrating a variety of sensory self-motion signals, we included a triangle completion task, involving both linear and rotational segments, and a whole-body rotation task, involving only rotations. There is evidence that the translational and rotational components of trajectories during path integration are processed separately to some degree [66], so these tasks, in concert with the target-directed and experimenter-guided walking tasks, offered a means of illuminating the role of the MTL in this possible functional separation. They were also similar to the tasks that had previously been tested on MTL-injured patients [47], [48], affording a comparison between the present study and the previous studies. To examine whether any motion signals other than those associated with walking would engage the MTL, we used a blind pulling task that involved integration of arm motions [67]. To investigate further the role of sensory self-motion signals for engaging MTL structures during integration tasks, we included two tasks that involved integration of body-related information with no physical body movement: imagined walking and third-person time-to-contact tasks, in which participants estimated how long it would take either themselves or another person, respectively, to reach a visible target by walking. Although some muscle and neural activation might be evoked by these tasks [68], such signals would be much more subtle than those evoked during overt body motion. These tasks thus provide a test of whether physical motion is required to elicit deficits in integration of body-related information after unilateral MTL resection. Finally, we included tests of spatial working memory, time perception, and lateralized spatial attention to rule out path integration impairments that might arise due to deficits in these factors. This allowed us to restrict our focus more narrowly on self-motion sensing and integration.

In this manner, by administering a large number of tasks in the same group of MTL-injured patients, the present study was designed to gain insight into the primary MTL roles for human path integration. By identifying tasks in which the patients exhibited impaired performance, we sought to determine component processes of path integration for which the integrity of the MTL is critical. Equally important was to identify tasks in which the patients did *not* show deficits, because this observation would help rule out possible secondary sources of impairment in path integration tasks. Given the relative rarity of the population of surgical epilepsy patients (particularly in light of the advent of neurostimulators as a new non-surgical treatment for medically intractable epilepsy), this study provided a unique opportunity to make a large-scale assessment of the effect of unilateral MTL damage on path integration. Data reported in this article would have significant value not only for building theories of the brain's path integration mechanisms, but also for adding to the empirical canon of tests administered to this rare population of patients.

## Materials and Methods

### Ethics statement

The protocol of this study was approved by the Institutional Review Board of the George Washington University. Participants gave written informed consent to participate in the study.

### Participants

#### Groups

Three groups of participants took part in the study (see Table 1). Two groups had undergone unilateral left- (*n* = 13) or right-hemisphere (*n* = 10) temporal lobe resection (LTLR and RTLR, respectively) as therapy for epilepsy; a third was the age-matched healthy control (CONT, *n* = 12) group who had no history of neurological disorder. An analysis of variance (ANOVA) showed no reliable differences in age between groups, *F*_(2, 30)_ = .21, *p* = .812, *η* ^2^ = .01. Exact ages were not available for two CONT participants, but informal observation suggested that their ages were near the mean of the group. The patient groups did not differ in terms of when testing was conducted relative to the temporal lobe resection, *t*_(22)_ = .59, *p* = .561. Participants were paid $10/hour (with testing session lasting 2–3 hours).

**Table 1:** Demographic details, neuropsychological test results, and lesion analyses of the participants^a^.

	CONT^b^	LTLR^b^	RTLR^b^
**Sex (M/F)**	5/7	4/9	6/4
**Age**	46 (30–58)	47 (29–63)	44 (31–58)
**Time test** c	N/A	5.9 (.5–19.6)	4.5 (.1–18.7)
**WMS-III LM I** d (max = 75)	35 (30–41)^e^	27 (13–46)	34 (12–48)
**WMS-III LM II** d (max = 50)	22 (14–27)^e^	12 (3–24)	20 (0–30)
**ROCF** f **copy** (max = 36)	31 (19–36)	33 (30–36)	28 (23–35)
**ROCF** f **delay** (max = 36)	16 (5–26)	13 (8–23)	10 (1–19)
**BIT** g (max = 105)	103 (100–105)	104 (103–105)^h^	103 (97–105)^i^
**Spatial span** j (max = 32)	15 (9–21)	15 (10–20)^k^	14 (9–20)
**Digit span** j (max = 30)	16 (13–21)	18 (13–25)	17 (12–22)
**Turn direction (L/R)** l	6/5	5/7	6/4
**STG resected** m	N/A	.5 (0–2.5)	1.7 (0–4)^n^
**MTG resected** m	N/A	2.2 (1–3)	2.7 (2–4)^n^
**ITG resected** m	N/A	2.6 (1.5–4)	2.6 (2–3)^n^
**HF resected** o	N/A	78 (70–80)	79 (70–80)
**PG resected** o	N/A	88 (80–90)	90 (all cases)
**Total TL resected** o	N/A	31 (20–40)	38 (30–40)

a Except as noted, mean values are presented, with the range in parentheses.

b CONT =  age-matched healthy control; LTLR =  left temporal lobe resection; RTLR =  right temporal lobe resection.

c Time of testing, post-surgery (years).

d Wechsler memory scale third edition [75], logical memory subtests (total score).

e *n* = 11.

f Rey-Osterrieth complex figure test [76].

g Behavioral inattention test [77].

h *n* = 10.

i *n* = 9.

j Wechsler memory scale third edition [75], total score.

k *n* = 12.

l Number of participants in each group who were exposed to leftward (counterclockwise) versus rightward (clockwise) body rotations in the triangle completion and whole-body rotation trials.

m Length of the superior temporal gyrus (STG), middle temporal gyrus (MTG), and inferior temporal gyrus (ITG) resected in cm, based on intraoperative measurements made in an anterior-to-posterior direction.

n *n* = 6.

o Estimate of the percentage of the hippocampal formation (HF), parahippocampal gyrus (PG), and total temporal lobe (TL) resected based on post-surgical brain images.

Given that the patients evaluated in the present study had intractable temporal lobe epilepsy and had a temporal lobectomy to treat that disorder, non-surgical epilepsy patients might seem to be more appropriate as a control group, particularly those having localized seizure foci lying outside the MTL and those diagnosed with generalized epilepsy with no clear seizure focus. By comparing surgical and non-surgical epilepsy patients, differences related to epilepsy per se might potentially be controlled across groups, thus permitting a more precise attribution of any behavioral group differences to the resected MTL structures. In practice, however, a non-surgical epilepsy group is less informative for control purposes than might initially be supposed. There is often epileptiform activity in MTL structures even in generalized seizures or when the seizure focus itself lies outside the MTL [69], and such abnormal neuronal activity very likely impacts MTL functions [70], [71]. Moreover, non-surgical patients generally take a greater amount of antiepileptic drugs than surgical patients [72], and many of these drugs are known to impair MTL functioning to a certain extent [73]. As a consequence, it is not clear whether the non-surgical epilepsy patients should be expected to exhibit normal or impaired performance in the tasks of the present study, making interpretation of the data from these patients equivocal. In light of these considerations, neurologically intact age-matched individuals were recruited for the control group in the present study.

#### Inclusion criteria

Participants were included in the study if they met the following criteria: age 18–75 years, 12 or more years of education, absence of dementia or psychiatric disorder, visual acuity at least 20/100 (corrected if necessary), and ability to walk well without assistance. Additional inclusion criteria for the patient groups included well-controlled seizures at time of testing, left hemisphere language dominance (based on pre-surgical sodium amobarbital testing), no neurological problems other than epilepsy, and no structural abnormalities outside the MTL.

#### Medial temporal lobectomy procedure

All but two surgeries were performed by AJC (the eighth author of this paper). The procedure resected anterior and lateral surfaces of the temporal lobe, amygdala, anterior hippocampus, and anterior parahippocampal gyrus [74]. To begin, a frontal-temporal craniotomy exposed the temporal lobe and a small portion of the frontal lobe for placement of an electrocorticography recording array. This array was used to map and define the epileptogenic area, occasionally with the aid of electrical or chemical (methohexital 20 mg) stimulation. Based on this mapping, portions of the anterior and lateral surfaces of the temporal lobe were resected until the area was no longer epileptogenic. Next, the amygdala and anterior portions of the parahippocampal gyrus and hippocampus were resected, sparing the fornix. Resection measurements were made when resection was determined to be complete from an electrocorticographic standpoint, starting from the temporal pole and measuring in an anterior-to-posterior direction. These intraoperative measurements indicated that 3 cm of the anterior hippocampus was removed in all patients for whom such measurements were available (intraoperative measurements were not available for four RTLR patients). In this procedure, it is standard for somewhat less of the lateral temporal lobe to be resected in the language-dominant hemisphere. To assess this quantitatively, we added the intraoperative resection measurements along the superior, middle, and inferior temporal gyri. The resulting sums averaged 5.12 and 7.08 cm for the LTLR and RTLR patients, respectively (Table 1). This difference was reliable, *t*_(18)_ = 2.35, *p* = .030. As an additional check, lesion volumes were estimated on the basis of post-operative brain images (obtained by computerized tomography or magnetic resonance imaging). Scans were available for all patients, thus providing a means of verifying that all patients (i.e., even those for whom intraoperative measurements were not available) had undergone surgical resections of approximately the same magnitude. Two neuroradiologists (one of them being LL, the seventh author) independently estimated, by visual inspection, the percentage of resected tissue relative to the intact volume on the contralesional side of each patient's brain. The two readings were averaged for each patient, separately for the hippocampal formation, parahippocampal gyrus, and the temporal lobe as a whole (Table 1). These analyses suggest that the resections for each group were highly similar overall; *t*-tests performed on these data showed no hemispheric differences in the percentage of tissue resection in the hippocampal and parahippocampal gyrus regions (*t*_(22)_ = .88 and 1.73, *p* = .388 and .098, respectively). However, significantly less tissue was resected in the left temporal lobe overall (*t*_(22)_ = 2.54, *p* = .018), mirroring the results of the intraoperative measurement analysis.

#### Neuropsychological tests

Participants' general cognitive abilities were evaluated by standardized neuropsychological tests (Table 1). They included (a) the logical memory (LM) I (immediate recall) and II (delayed recall and recognition) subtests (total score measures) of the Wechsler memory scale third edition (WMS-III) [75]; (b) the copy and delayed recall measures of the Rey-Osterrieth complex figure test (ROCF) [76]; (c) the conventional subtest of the behavioral inattention test (BIT) [77], commonly used to detect biases in directing attention to the left versus right hemispace; and (d) the forward and backward versions of the spatial span and digit span tests of the WMS-III (total score measures), which provide measures of working memory.

### Design and apparatus

Each participant completed all tasks in a single session. Except as noted, each task was completed in a 6×6 m laboratory or in a 1.8×10 m hallway. Participants were exposed to one practice trial for each task without error feedback to familiarize them with the procedure. The following sequence of tests within each session was used: triangle completion, blind pulling, imagined walking, target-directed walking, whole-body rotation, verbal distance estimation and delayed distance matching, experimenter-guided walking, time estimation, and third-person time-to-contact. This task order was designed to increase participants' engagement and prevent fatigue during the experiment by intermixing tasks in which participants had to stand on their feet with those in which they were seated. Prior to each trial, participants were given a four-digit number, which they attempted to recall immediately after executing their response. A new number was randomly generated for each trial. Because participants often retain this information via rehearsal, this concurrent task was intended to discourage sub-vocal pace-counting during walking trials; it was implemented in other trials to maintain consistency across other trial types. We did not analyze the data as a function of accuracy in recalling the memory number, although memory number recall was generally good for all groups. Except as noted, visual targets were presented at several possible absolute distances: 2.5 and 5 m were presented three times apiece; targets at 1.5, 3.5 and 5.5 m were included to increase the range and variability of the stimulus distances, but were measured once apiece to minimize the total number of trials. The same stimulus distances were used in tasks in which visual targets were not presented (e.g., experimenter-guided walking). The resulting nine trials were presented in random order. Viewing was binocular. In triangle completion and whole-body rotation, the body rotations were always in the same direction for each participant across these tasks; approximately half of the participants in each group always turned to the left, while the rest always turned to the right (see Table 1).

### Procedure

#### Target-directed walking

Participants viewed a 23 cm tall orange cone placed on the floor, then lowered a blindfold and attempted to walk unaided to the remembered target location without vision. The same cone was used as a visual target in other tasks unless otherwise noted. An assistant removed the target before participants began to walk. After stopping, participants were guided back to the starting location without vision and without knowledge of results.

#### Experimenter-guided walking

Participants began each trial by viewing an empty hallway, then lowered a blindfold and held an experimenter's arm for support. The experimenter walked with the blindfolded participant along a straight path. Upon stopping, participants gave a verbal estimate of the walked distance. They were then guided back to the starting location without vision and without knowledge of results.

#### Verbal distance estimation and delayed distance matching

These trials were conducted with participants standing at the intersection of two perpendicular hallways. Participants viewed the target in one hallway and gave a verbal estimate of its distance. After a delay of approximately 5 s, during which the target was removed, the experimenter verbally cued participants to turn to face the second hallway. There, they saw an identical cone placed 6.5 m away. An experimenter started to move this cone toward the participant, who instructed the experimenter to stop when the cone's distance appeared to match that of the target seen from the starting position in the first hallway. Participants then turned to face the first hallway for the next trial.

#### Triangle completion

While holding onto the experimenter's arm for support, blindfolded participants were guided along two legs of a triangle, separated by a whole-body rotation. At the end of the second leg, participants attempted to walk back to the origin of locomotion. Participants were guided along a short, circuitous path between trials to avoid providing error feedback about performance on the previous trial. Vision of the environment was provided between trials. The first straight segment of each path was 1.5 m; this was followed by a turn of 30° or 110°, and then a second straight segment of 1 or 2 m. Each of the resulting four possible paths was repeated three times in random order.

#### Whole-body rotation

Participants sat in a chair and underwent passive, whole-body rotations of 30°, 75° or 120° (three times apiece in random order). Vision of the environment was provided before each body rotation, but the rotations themselves were administered without vision. The rotations were delivered using a computer-controlled device described elsewhere [78]. The velocity profile was roughly triangular, and consisted of an initial acceleration of 90°/s^2^ up to a peak velocity of 54, 81, and 90°/s for the 30°, 75°, and 120° rotations, respectively. The accelerating phase was followed immediately by a deceleration at the same rate for the 30° and 75° rotations; there was a brief period at constant velocity for the 120° rotations before the decelerating phase. A pointing device was anchored to the participants' chair just in front of their abdomen. The experimenter aimed the pointer toward the participants' abdomen before each trial. After the body rotation, the blindfolded participants manipulated the pointer with their right hand and attempted to aim it at a specified origin, located straight ahead before the rotation and 76 cm from the rotation axis of the chair. After pointing, participants were returned to the starting orientation using the same velocity profile in reverse.

#### Imagined walking

With eyes open, participants first physically walked to targets at 2.5 and 5 m, three times apiece in random order, under the instruction to walk using a “normal” pace. An experimenter recorded these walking times using a stopwatch. These walking trials were conducted just prior to the triangle completion trials in the sequence of testing. Approximately 20 minutes later, participants again viewed targets either 2.5 or 5 m away, then closed the eyes and imagined themselves walking to the target using the same normal pace. They started and stopped a stopwatch to mark out the duration of the imagined walk. As before, each distance was presented three times apiece in random order. The task was well-matched to target-directed walking in terms of memory load, viewing environment, and in the spatial and temporal intervals that must be processed. However, imagined walking provided virtually no sensory motion signals.

#### Blind pulling

Participants sat in a chair and held one end of a measuring tape, with the experimenter holding the other end on the other side of the laboratory, keeping the tape relatively taut and parallel to the floor. A pipe cleaner wrapped around the tape was used to indicate the target distance. After viewing the pipe cleaner, participants lowered a blindfold and the pipe cleaner was removed. Participants then used hand-over-hand motions to draw the tape toward them until they felt that the amount of tape pulled matched the viewed target distance. More details of this task are available elsewhere [67].

#### Third-person time-to-contact

On a computer monitor, participants saw a video showing a man walking in place on a treadmill. The man's image appeared on the right-hand edge of the screen, with the man facing toward the left. At a viewing distance of 75 cm, the image subtended 3.06° of visual angle. The man's pace was either .9 or 1.8 m/s. The walking motions provided information about the man's walking velocity, but the net optical velocity was zero, because the man remained in place while walking and the image did not progress across the computer monitor. After several paces (yielding videos of 2.5 or 1.5 s in duration for the .9 and 1.8 m/s walking paces, respectively), the video disappeared and a vertical line appeared, situated such that it would rest on the same ground plane as the man. Participants pressed a button to indicate when the man would reach the line if he had started to move across the screen at the observed pace. The line appeared at distances corresponding to 2.88 or 9.14 m at the scale of the man's height in the video. Each combination of walking velocity and target distance was presented four times in random order. This task was well-matched to target-directed walking, in terms of time scale, memory load, and the requirement of integrating motion over time. It required integrating visual information about biomechanical motion to determine speed, and then using this to estimate time to contact. It provided little or no idiothetic self-motion signals, however.

#### Time estimation

Participants sat in front of a computer monitor and verbally estimated the time interval that elapsed between two white flashes on the monitor. Three time intervals (2, 5, and 8 s) were presented three times apiece in random order.

### Data analysis

Responses were converted to signed (constant) errors, expressed as a percentage of the correct values. The signed errors provide a measure of the degree to which responses tended to overestimate or underestimate the correct value. Unless otherwise noted below, the signed errors in each task were averaged across repetition and stimulus dimensions (e.g., walked distances). One way of analyzing these data would be to run an omnibus ANOVA with group (CONT, LTLR, and RTLR) as a between-subject factor and task (target-directed walking, experimenter-guided walking, etc.) as a within-subject factor, and examine the interaction between the two variables. However, because we employed a large number of tasks, there would be numerous patterns of data that could make the interaction significant but did not pertain to the hypotheses of the present study. Therefore, we opted for examining group differences more directly by running separate one-way ANOVAs for each task in which group was a between-subject factor. Any main effects of group were followed by pairwise group comparisons using the Tukey-Kramer honestly significant difference test.

All the data were also analyzed without averaging by mixed ANOVAs in which respective stimulus dimensions constituted additional within-subject factors. Data from certain trials were omitted from these analyses as necessary, because not all levels of the within-subject factors were measured equally (e.g., target distances of 1.5, 3.5, and 5.5 m were used only once apiece). None of these analyses altered the findings reported below.

We also analyzed unsigned (absolute) errors and within-subject variable errors in the same manner as signed errors. However, these analyses did not yield any significant differences between participant groups. Therefore, participants' performance was examined in detail in terms of signed errors in this article. Unsigned and variable errors are reported in Supporting Information (Text S1, Table S1, and Table S2).

## Results

### Neuropsychological tests

Participants' performance on the neuropsychological tests was compared in separate ANOVAs. LM I and II data were not obtained for one CONT participant, BIT data were not obtained for three LTLR and one RTLR participants, and spatial span data were not obtained for one LTLR participant. Group means and ranges of scores are reported in Table 1.

There were group differences for the LM II but not the LM I test. Statistical results for the LM I and LM II tests, respectively, were *F*_(2, 31)_ = 2.05 and 6.55, *p* = .146 and .004, *η* ^2^ = .12 and .30. Pairwise contrasts for LM II showed that the LTLR group performed more poorly than the CONT and RTLR groups (*p* = .002 and .012, respectively), but that the CONT and RTLR groups did not differ from each other (*p* = .571). In the copy portion of the ROCF, there was a group effect (*F*_(2, 32)_ = 3.80, *p* = .033, *η* ^2^ = .19). Pairwise contrasts showed that the LTLR group performed reliably better than the RTLR group (*p* = .010), and that the RTLR group performed marginally worse than the CONT group (*p* = .078). The LTLR and CONT groups did not differ significantly (*p* = .364). The groups also differed reliably in the delayed recall portion of the ROCF (*F*_(2, 32)_ = 3.96, *p* = .029, *η* ^2^ = .20): RTLR patients performed more poorly than the CONT participants (*p* = .008), with no significant difference between either LTLR and RTLR (*p* = .094) or CONT and LTLR (*p* = .243). There were no group differences in the BIT (*F*_(2, 28)_ = 1.28, *p* = .294, *η* ^2^ = .08). Similarly, there were no main effects of group for the spatial and digit span tests (*F*_(2, 31)_ = .22 and *F*_(2, 32)_ = 1.73, respectively; both *p*s>.193, *η* ^2^s<.10).

These tests show that the patient groups exhibited normal visuospatial attention (as indicated by the BIT) and working memory function (as indicated by the spatial and digit span tests). As is typical [49], the patient groups exhibited some deficits in longer-term memory, with the LTLR group scoring somewhat more poorly on the LM test of verbal memory and the RTLR group scoring relatively poorly in the more spatial ROCF test.

### Behavioral tests

Mean signed errors for each group are presented in Table 2, along with their standard errors and results of statistical tests for the main effect of group in each task. We also computed correlations between mean signed errors and the time of testing relative to surgery to examine possible effects of this variable on participants' performance. However, these correlations were small and none of them was reliably different from zero (−.30<*r*s<.30, *p*s>.185).

**Table 2:** Mean signed errors of the three participant groups and results of statistical analyses for each task.

		Group means (and their standard errors)^a^		
Task	*F*, *p*, and *η* ^2^ statistics^b^	CONT^c^	LTLR^c^	RTLR^c^
**Target-directed walking**	*F*_(2, 32)_ = 3.35, *p* = .048, *η* ^2^ = .17	−8.94 (3.09)	1.94 (4.29)	6.79 (5.64)
**Experimenter-guided walking**	*F*_(2, 32)_ = 2.17, *p* = .131, *η* ^2^ = .12	−23.86 (7.27)	−4.93 (10.24)	−27.93 (6.07)
**Verbal distance estimation**	*F*_(2, 32)_ = 1.46, *p* = .246, *η* ^2^ = .08	−19.89 (5.28)	−1.74 (10.76)	−15.13 (5.17)
**Delayed distance matching**	*F*_(2, 31)_ = .66, *p* = .523, *η* ^2^ = .04	−.38 (3.03)	4.12 (3.73)	−1.07 (3.71)
**Triangle completion**				
**Response turn**	*F*_(2, 32)_ = 1.15, *p* = .328, *η* ^2^ = .07	3.93 (4.30)	1.81 (3.71)	−4.86 (4.30)
**Response leg**	*F*_(2, 32)_ = 1.29, *p* = .290, *η* ^2^ = .07	7.16 (4.66)	17.10 (4.40)	15.44 (5.41)
**Stopping point error** d	*F*_(2, 32)_ = .26, *p* = .771, *η* ^2^ = .02	.99 (.09)	.96 (.10)	1.07 (.11)
**Whole-body rotation** e	*F*_(2, 30)_ = .42, *p* = .659, *η* ^2^ = .03	1.84 (8.59)	−2.93 (5.77)	−8.22 (7.89)
**Imagined walking** f	*F*_(2, 32)_ = .51, *p* = .602, *η* ^2^ = .03	.52 (5.49)	11.80 (12.85)	17.01 (14.92)
**Blind pulling**	*F*_(2, 32)_ = 1.06, *p* = .357, *η* ^2^ = .06	−15.61 (6.02)	−2.84 (13.51)	−23.29 (6.73)
**Third-person time-to-contact**	*F*_(2, 32)_ = 1.02, *p* = .371, *η* ^2^ = .06	−11.32 (11.49)	−3.64 (15.58)	18.87 (17.37)
**Time estimation**	*F*_(2, 31)_ = .87, *p* = .429, *η* ^2^ = .05	30.65 (9.90)	40.39 (19.85)	13.05 (9.89)

a Except as noted, group means are expressed as a percentage of the correct response values.

b Statistics associated with the test of the main effect of group in each task. Degrees of freedom are not uniform across the tasks because some participants were not tested in all of the tasks. For details, see the results section of the text.

c CONT =  age-matched healthy control; LTLR =  left temporal lobe resection; RTLR =  right temporal lobe resection.

d Mean straight-line distances (m) to the correct stopping point (i.e., the origin).

e The data reported in this table were corrected for possible errors in response execution by following the procedure described in the results section of the text. Uncorrected data are shown in Supporting Information (Table S3).

f Differences between mean imagined walking time and mean real walking time expressed as a percentage of the mean real walking time. For details, see the results section of the text.

#### Target-directed walking

**Figure 1:**
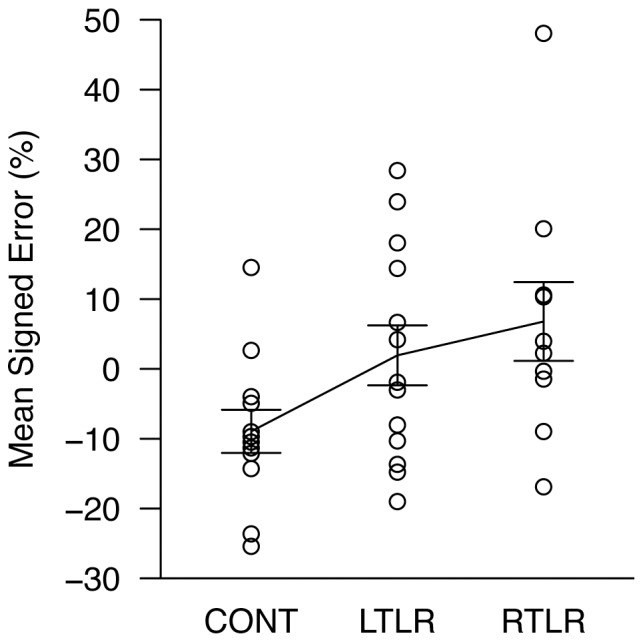
Mean walked distances in target-directed walking, expressed as a percentage of the target distance. Each data point is the mean response for one participant, collapsed over nine measurements; data for all three participant groups are shown (CONT =  age-matched healthy control; LTLR =  left temporal lobe resection; RTLR =  right temporal lobe resection). The solid line indicates the mean level of performance for each group. Error bars represent ±1 standard error of the mean.

There was a main effect of group (Table 2). A pairwise contrast showed that the RTLR group walked significantly farther than the CONT group (*p* = .018). The LTLR group exhibited a similar trend (CONT vs. LTLR: *p* = .075), and the two patient groups did not differ reliably from each other (*p* = .442). This pattern of data is more clearly seen in Figure 1.

#### Experimenter-guided walking

**Figure 2:**
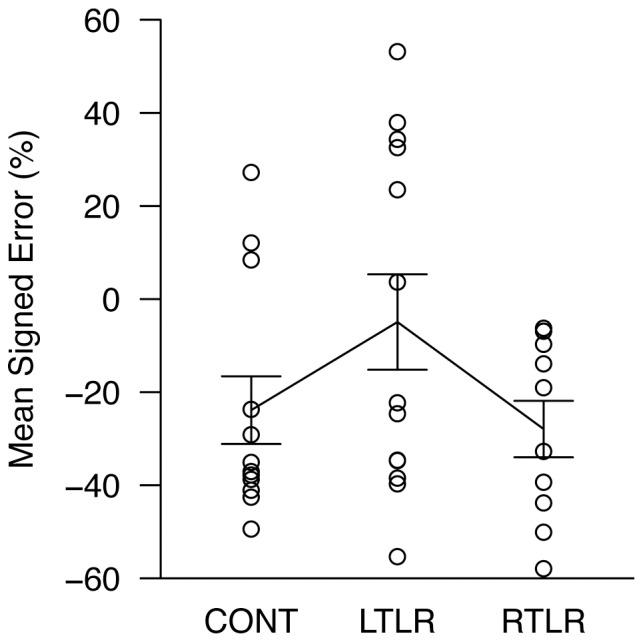
Mean distance estimates in experimenter-guided walking, expressed as a percentage of the stimulus distance. Each data point is the mean response for one participant, collapsed over nine measurements; data for all three participant groups are shown (CONT =  age-matched healthy control; LTLR =  left temporal lobe resection; RTLR =  right temporal lobe resection). The solid line indicates the mean level of performance for each group. Error bars represent ±1 standard error of the mean.

Mean signed errors for each participant are plotted in Figure 2, showing that there was more spread among LTLR than RTLR patients in this task. However, there was substantial overlap across the three groups, and statistical comparisons revealed no significant group differences (Table 2). Although there might be small group differences in the experimenter-guided walking trials that were obscured by within-group variability, the pattern of data was distinctly different than in the target-directed walking trials, in which there was clear shift in the distributions of the two patient groups relative to the CONT group.

#### Verbal distance estimation and delayed distance matching

**Figure 3:**
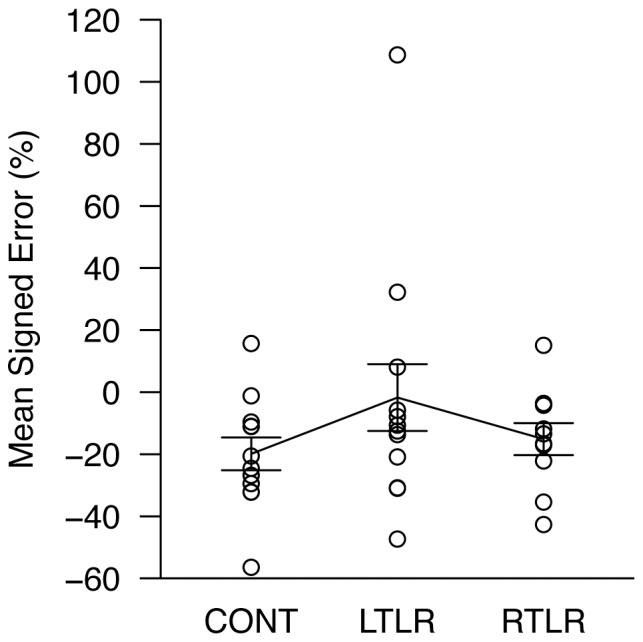
Mean indicated distances in verbal distance estimation, expressed as a percentage of the target distance. Each data point is the mean response for one participant, collapsed over nine measurements; data for all three participant groups are shown (CONT =  age-matched healthy control; LTLR =  left temporal lobe resection; RTLR =  right temporal lobe resection). The solid line indicates the mean level of performance for each group. Error bars represent ±1 standard error of the mean.

**Figure 4:**
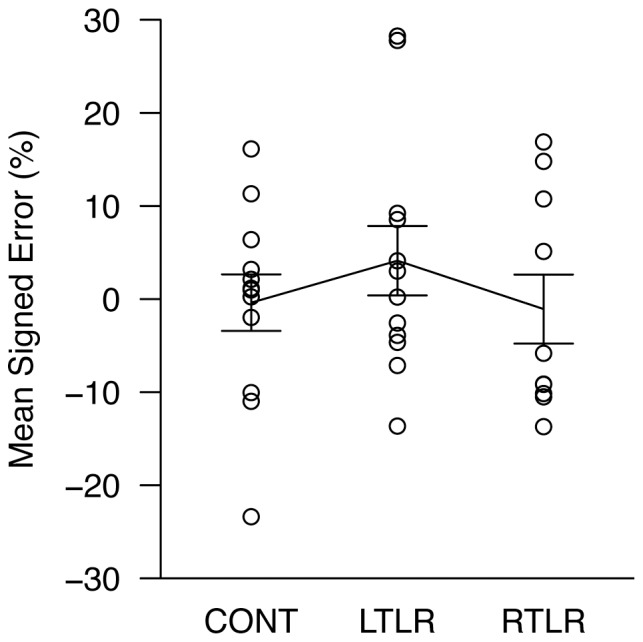
Mean indicated distances in delayed distance matching, expressed as a percentage of the target distance. Each data point is the mean response for one participant, collapsed over nine measurements; data for all three participant groups are shown (CONT =  age-matched healthy control; LTLR =  left temporal lobe resection; RTLR =  right temporal lobe resection). The solid line indicates the mean level of performance for each group. Error bars represent ±1 standard error of the mean.

The delayed distance matching task was not administered to one LTLR patient. No reliable group differences were found in these tasks (see also Figures 3 and 4 for mean signed errors of individual participants).

#### Triangle completion

We calculated the *response turn* and *response leg* on each trial, which were the body rotation and final path length that participants generated when attempting to walk back to the origin. These were calculated relative to the participants' location and heading at the “drop off” point–i.e., at the end of the second leg of the triangular path. The response turns and legs were compared with the corresponding values for an accurate response path, and these error scores were used to generate signed errors as a percentage of the accurate path values. We also calculated the *stopping point error* for each response, which was the straight-line distance of the actual stopping point from the ideal stopping point (the origin). Overall group means and more detailed means per each triangular path are presented in Tables 2 and 3, respectively. No group differences were found in any of these analyses.

**Table 3:** Mean signed errors in triangle completion trials for the three participant groups.

		Group means (and their standard errors)^a^		
Response parameter (by stimulus path)	Correct response value	CONT^b^	LTLR^b^	RTLR^b^
**Response turn**				
1 m, 30°	109°	49 (5.6)	47 (4.2)	37 (7.6)
1 m, 110°	136°	−16 (5.3)	−17 (5.4)	−21 (5.3)
2 m, 30°	161°	2 (3.3)	−1 (3.5)	−7 (3.1)
2 m, 110°	167°	−19 (4.7)	−21 (3.9)	−28 (3.0)
**Response leg**				
1 m, 30°	1.49 m	57 (6.7)	73 (8.1)	68 (8.4)
1 m, 110°	2.05 m	−9 (5.4)	−8 (5.4)	−9 (5.7)
2 m, 30°	2.42 m	14 (5.1)	32 (6.8)	36 (6.9)
2 m, 110°	3.38 m	−33 (4.1)	−29 (3.3)	−33 (3.9)
**Stopping point error**				
1 m, 30°	0	.9 (.1)	.8 (.1)	1.1 (.2)
1 m, 110°	0	.9 (.1)	.9 (.1)	.8 (.1)
2 m, 30°	0	1.2 (.1)	1.2 (.2)	1.4 (.2)
2 m, 110°	0	1.0 (.1)	1.0 (.1)	1.0 (.1)

a For response turn and response leg, group means are expressed as a percentage of the correct response values. For stopping point error, because the correct response value is zero, actual mean distances to the correct stopping point (i.e., the origin) are shown in meters.

b CONT =  age-matched healthy control; LTLR =  left temporal lobe resection; RTLR =  right temporal lobe resection.

#### Whole-body rotation

This test was not administered to one LTLR patient. In addition, data from one CONT participant were omitted, as the responses were not obviously related to the body rotation magnitudes and thus likely indicated confusion about the task requirements. We used linear statistics to analyze the data, but means and angular deviations calculated by circular statistics [79] showed the same results.

Three participants (one in each group) occasionally pointed to the incorrect side of the left-right body axis. Because these cases were observed in all participant groups in equal frequency and were often highly consistent with the participant's other responses to the same body rotation when flipped about the left-right axis, it was likely that these wrong-side responses were due to simple errors in response execution that occurred independently of the effect of MTL injury. Thus, we dealt with these responses by flipping them about the left-right axis (e.g., a response of 40° to the left was transformed to 40° to the right). One additional RTLR participant appeared to be aiming the rear end of the pointer toward the origin rather than the front, resulting in several wrong-side responses. We corrected for these errors before data analysis, and results are shown in Table 2. We also analyzed the raw data to verify that our transformations related to wrong-side pointing errors did not introduce any significant bias in the data. The raw data are reported in Supporting Information (Table S3). The participant groups did not differ significantly in any of these analyses.

#### Imagined walking

We first assessed whether the groups differed in terms of their physical walking rates. We did this by dividing the target distance by the time required to physically walk to the target, and then averaging the resulting walking rates across target distances. The groups did not differ (*F*_(2, 32)_ = .31, *p* = .736, *η* ^2^ = .02), with the physical walking rate averaging 1.06 m/s. To assess *imagined* walking, we calculated each participant's average real walking time for each target, and then subtracted their mean *real* walking time from the *imagined* walking time. We expressed this difference score as a percentage of the mean real walking time and averaged across target and repetition prior to analysis. No group differences were found in this measure either, as shown in Table 2.

#### Blind pulling, third-person time-to-contact, and time estimation

No group differences were found in these tasks.

## Discussion

### Summary of major findings

In this study, we tested the MTL's role in path integration using a variety of tasks. There was a reliable difference between patients and controls in the target-directed walking task, with the RTLR patients walking significantly farther than the CONT participants. The LTLR patients also walked more than the controls, although the difference between LTLR and CONT groups did not reach statistical significance (*p* = .075). Importantly, overshooting in this task was not due to over-perception of the target's distance prior to walking, as indicated by the patients' normal verbal distance estimates and normal delayed matching responses relative to the controls. Similarly, deficits in time perception, working memory, and the general ability to integrate motion over time were not responsible, as there were no group differences in time estimation, delayed distance matching, imagined walking, blind pulling, and third-person time-to-contact tasks (see also Table 1 for their spatial span and digit span data). Furthermore, impairment in target-directed walking was likely not due to general effects of brain injury, as patients with parietal damage have been shown to perform this task normally [52]. Together, these results strongly implicate path integration as the source of the group differences in target-directed walking. This is a significant advancement in the investigation of the MTL's role in human path integration because no studies to date have ruled out this many possible secondary sources of impairment underlying the observed path integration deficits [47]–[49].

One more notable finding of the present study is that there was no reliable difference between patients and controls in the experimenter-guided walking task. Given the clear dissociation between the patients (especially RTLR patients) and controls in the target-directed walking task, the lack of group differences in the experimenter-guided walking task might appear inconsistent: both tasks involve visual information about the surrounding environment and idiothetic signals arising from linear self-motion. As discussed in the introduction, an important difference between experimenter-guided walking and target-directed walking is that only the target-directed walking task allows participants to form a mental representation of the environment including the target and begin their walking response with an expectation about the magnitude and trajectory of their upcoming locomotion. There is evidence that MTL structures play a role in constructing spatial representations [12]–[16] and predicting an upcoming locomotion trajectory [53]. In addition, it has been shown that having access to this kind of expectation about upcoming locomotion can improve path integration [59], [60]. Together, it is possible that the patients did not fully draw the benefit of trajectory predictions in target-directed walking trials due to disruption of expectation-related signals in the MTL, accounting for the behavioral dissociation in the patient groups. These data suggest that the MTL's contribution to human path integration comes not merely through a role in processing idiothetic self-motion signals per se, but via a role in generating or monitoring signals that predict the consequences of one's upcoming locomotor actions.

Aside from the fact that upcoming walking trajectories were predictable only in target-directed walking, this task also differed from experimenter-guided walking in response methods: target-directed walking involved a motoric response (i.e., blind walking), whereas experimenter-guided walking utilized verbal estimation of walked distance. It is unlikely, however, that these response methods were the primary factors that modulated group differences in these tasks. In our test battery, there were other tasks that used motoric responses (i.e., triangle completion, whole-body rotation, and blind pulling), but no group differences were found from them. Similarly, participants verbally estimated distance in the verbal distance estimation task, but the highly consistent pattern of responses across all participant groups suggests that the use of verbal estimation per se did not mask any possible group differences (see Figure 3). Thus, the critical difference that remains between target-directed walking and experimenter-guided walking is the predictability of upcoming walking trajectories, to which the observed dissociation of patient performance between the two tasks is attributed.

The group differences in the target-directed walking task were such that the control group tended to undershoot, whereas the patient groups walked farther than the control group. This had the effect of making the patient groups' responses somewhat more accurate than the control participants', relative to the physical target locations (see Figure 1). This may seem counterintuitive (i.e., brain injury resulting in better-than-normal performance), but it should be noted that absolute accuracy is not as meaningful as differences relative to the control group. For one thing, there are good reasons to expect that undershooting in this task indeed represents the peak performance typically achieved by neurologically intact observers. Past work has shown that absolute accuracy in this task varies according to various environmental and experimental variables such as the size of an enclosing space and the number of blind walking trials in an experiment [80]. Undershooting in this task has been found when a small number of trials were performed in small indoor spaces [80], [81], which was the case in the present study. As such, the control group's undershooting performance is the appropriate basis of comparison for the patient groups. Second, the lack of group differences in other tasks rules out a variety of possible factors that might otherwise contribute to increased accuracy in this task. Our interpretation is that the patients were under-perceiving their self-motion in this task, relative to the level of self-motion perception in the control group. That is, in order to walk to the perceived target location, they had to walk a bit farther, relative to the controls, to counteract their underestimation of self-motion.

As noted above, our results suggest that prediction of upcoming locomotion paths is a viable function underlying the dissociation we observed in MTL-injured patients. In this view, MTL structures tend to be engaged in path integration tasks to the extent that the task allows individuals to anticipate their own locomotor actions. The upcoming walking trajectory is clearly available in our target-directed walking task. Arguably, however, certain other tasks in our battery also afforded at least some element of trajectory prediction. In the blind pulling task, participants previewed a target location, and this could allow them to establish a prediction about the effect of their arm movements on the trajectory of the target before they began moving their arms. In the third-person time-to-contact task, participants saw a target and had to predict the effect of another person's locomotion to judge when the actor would reach the line. In the imagined walking task, participants saw a target and again had to predict the effect of their walking in terms of the travel time required to reach the target. Nevertheless, we found no MTL-related performance differences in any of these tasks. The null effects in these tasks suggest three possibilities. First, these results might demonstrate that the mere presence of prediction-related processing is not sufficient to recruit the MTL during self-motion updating–the patients remained able to predict the consequences of certain kinds of actions. Instead, the MTL might be engaged during path integration more specifically when the task affords predicting the consequences of one's physical locomotion through space. Second, it is possible that predictions formed in blind pulling, third-person time-to-contact, and imagined walking tasks were not very accurate or precise because participants most likely had little or no accumulated experience with these tasks (compared to non-visual walking), and this lack of sufficient accuracy or precision in the predictions prevented them from exerting observable effects on participants' performance in these tasks. In other words, the degree of MTL engagement during path integration may depend upon the fidelity of prediction-related signals. Third, the prediction-related signals formed in these tasks might have been weaker than those present in the target-directed walking task (perhaps due to one or both of the reasons mentioned above), and therefore behavioral deficits in these tasks might require more pronounced damage to the MTL than that in our sample (e.g., bilateral MTL injury; note that our patients had unilateral lesions). These possibilities should be further explored in future studies.

Another objective of this study was to investigate whether the MTL plays a general role in integrating self-motion signals without regard to the sensory modality generating those signals, or instead preferentially processes information from some modalities. We found no differences between patients and controls in blind pulling and whole-body rotation, tasks targeting information from arm motion and the semicircular canals, respectively. However, the patients did differ from controls in target-directed walking, a task involving leg motion and otolithic signals. Thus, at a behavioral level, there appears to be some degree of modality-specific processing, with integration of arm motions and rotational vestibular signals not relying crucially on the integrity of MTL structures. However, there were no group differences in experimenter-guided walking and triangle completion, which generated motion signals from most or all of the same sensory modalities as target-directed walking. As such, the mere presence of self-motion signals from certain sensory modalities is not the only relevant factor determining the MTL's involvement in path integration.

### Relevance of the present findings to previous studies

Results from target-directed walking and experimenter-guided walking tasks were very similar to those obtained from the same tasks in our previous work [49]. Specifically, in both studies, the RTLR group walked significantly farther than the CONT group in target-directed walking, whereas these groups did not differ reliably in experimenter-guided walking. These converging findings demonstrate the reliability of the behavioral dissociation between these tasks shown by the MTL-injured patients who had lesions in the right hemisphere. Furthermore, these patients repeatedly yielded intact performance in verbal distance estimation and delayed distance matching tasks across the two studies, corroborating that the dissociation between target-directed walking and experimenter-guided walking was not confounded by possible impairments in visual perception and spatial working memory.

It is worth emphasizing that the successful replication of the original findings of Philbeck et al. [49] demonstrates the robustness of the patterns of data we discussed above. One might suspect that the significant group difference in the target-directed walking task was coincidental (i.e., Type I error), given the number of statistical tests conducted in the present study. However, it is very unlikely for two separate studies to yield the same main effect from one specific task by chance out of the large test battery. Furthermore, there was not merely a replication of the group main effect in the target-directed walking task across studies–the pattern of the effect across groups was also replicated (i.e., the RTLR group walked a longer distance on average than the CONT group). Taken together, the evidence suggests that the RTLR patients' tendency to walk farther in this task is highly reliable.

It should also be noted, however, that there were some differences between the present study and the previous study [49]. In the present study, both LTLR and RTLR groups walked farther than the CONT group in the target-directed walking task (see Figure 1), although the difference between LTLR and CONT groups was less distinct than that between RTLR and CONT groups. On the other hand, in the same task of the previous study, only the RTLR group was clearly different from the CONT group–performance of LTLR and CONT groups was indistinguishable from one another (see Figures 2A and 3 in [49]). In addition, the LTLR group of the previous study tended to underestimate distance in the experimenter-guided walking task relative to RTLR and CONT groups, and this tendency was observed to a lesser extent (i.e., as a non-significant trend) in the verbal distance estimation task as well. Overall, data from RTLR and CONT groups were highly consistent across the two studies, whereas those from LTLR groups were more variable. In line with this observation, Worsley et al. [48] have shown deficits in path integration tasks after right, but not left, medial temporal lobectomy. Similarly, Wolbers et al. [39] found that only the right hippocampal activation was associated with accurate performance in path integration based on optic flow. Moreover, generally, the right MTL shows advantage for processing spatial information over the left MTL [7], [10], [33], [35], [36], [82]–[84]. By contrast, evidence for the left MTL's involvement in path integration is not absent but sparse. For example, Sherrill et al. [38] showed that the left hippocampus was activated when observers performed path integration to navigate to a target whose location was previously specified in a map. These findings suggest that the right MTL plays a robust role in path integration, whereas the engagement of the left MTL in path integration tasks might be modulated by factors that are currently unknown. Further research is required to resolve this issue.

When participants are released at the end of the second leg in a triangle completion trial, they become able to predict their upcoming path back to the origin, and thus one might expect control participants to perform somewhat better than the patient groups. However, we found no group differences in this task. Similarly, in the Kim et al. [50] study, MTL-injured patients were unimpaired relative to controls when they attempted to come back to the origin of locomotion after walking along paths containing multiple linear segments and turns. In our triangle completion task, the response path on each trial was relatively small compared to the experimenter-guided portions, and this may have reduced the sensitivity of this task to prediction-related effects. This possibility is supported by evidence that normal triangle completion performance is enhanced when the proportion of actively controlled path segments is increased–presumably because participants are able to predict a larger proportion of their upcoming walking trajectory [59]. Nevertheless, the null results appear to conflict with those of Worsley et al. [48], who found that RTLR patients generated larger response turn errors in a triangle completion task than either LTLR patients or controls. One notable difference between the present study and the Worsley et al. study is that, in our study, the required response turn and leg varied from trial to trial. This was also the case in the Kim et al. [50] study. By contrast, in the Worsley et al. study, the origin and the release point were always the same. This likely boosted the control participants' ability to predict their upcoming response trajectory, relative to our study and the Kim et al. [50] study. Although participants in the Worsley et al. study had to generate a different response turn on each trial, they could come to anticipate the required heading relative to the environment (e.g., “turn South”). It is possible that the RTLR participants were unable to make use of this prediction-related benefit and therefore exhibited deficits relative to the other groups. The unchanging response leg length could have reduced the necessity of performing linear path integration at all, thereby eliminating group differences in this aspect of the response.

Worsley et al. [48] also included turn, distance, and route reproduction tasks, in each of which half of the trajectory could be predicted. Their RTLR patients were impaired relative to controls in the route reproduction task. There were no group differences in the turn and distance reproduction tasks, however. It is possible that the patients performed these tasks by matching the duration of the stimulus rotations and distances, rather than by performing path integration. Consistent with this idea, patients in the current study performed normally in the time estimation task.

Shrager et al. [51] tested five patients having varying degrees of bilateral MTL lesions on tasks that were conceptually similar to our triangle completion task (i.e., an experimenter led blindfolded participants along paths containing multiple straight segments and whole-body rotations, and participants then attempted to point to the starting position). The patients were unimpaired relative to age-matched healthy controls on these tasks–apparently contradicting the Worsley et al. [48] results. Shrager et al. argued that these results demonstrate that integrity of the MTL is not required for normal path integration (see also [50]). Regarding this apparent discrepancy between the Shrager et al. and Worsley et al. studies, it is important to point out that Shrager et al.'s tasks provided little opportunity for participants to predict their upcoming trajectory–not only because the entire trajectories were determined by the experimenter, but also because the response entailed pointing with the arm rather than actively producing locomotion. Under these circumstances, the MTL-injured patients and controls could be expected to perform similarly because the tasks did not elicit the benefit the controls would otherwise experience from being able to anticipate their upcoming trajectory.

In a separate study focusing on whole-body rotations [47], patients with unilateral hippocampal atrophy or sclerosis (especially those who had lesions on the right hemisphere) showed deficits in a task similar to our whole-body rotation task. It is notable that participants in this study made a response by rotating a chair back to the initial position. On the other hand, our participants responded by pointing to the origin of rotation with a pointing device. This difference between the tasks is important because only the task used in the previous study allowed participants to predict trajectories of their upcoming body movements. It is possible that the controls took greater advantage of this prediction than the patients did while moving back to the initial position, thereby creating the group difference.

Among the studies discussed above, some involved patients who had extensive damage to both anterior and posterior regions of the MTL [50], [51], while others (including the present study) tested patients whose lesions were primarily in the anterior MTL [48], [49]. Because this distinction did not make a clear separation of results between the two groups of studies, it remains to be seen whether the locus of injury within the MTL can lead to any behavioral dissociation in path integration performance [12], [85]–[87].

### Linkage between target-directed walking and long-term memory

Our primary interest in this study was performance in integration tasks involving relatively short (4–8 s) memory retention intervals–durations at which the patient groups' memory was not noticeably impaired. Nevertheless, it may be worth examining whether the patients' deficits in longer-term memory were linked with performance in these brief tasks, given that some patients showed relatively severe impairment in long-term memory (see Table 1). We focused here on the possible linkage between long-term memory and target-directed walking performance–the one task in our battery that showed clear group differences. We calculated Pearson correlation values between the unsigned errors in target-directed walking and scores in WMS-III LM II and ROCF (delayed recall) separately for LTLR and RTLR groups. We also computed the same correlation values by using scores in the copy portion of ROCF, although this test did not measure long-term memory. We included scores from this test in the analysis because RTLR and CONT groups showed a marginally significant difference. These *r* values ranged from −.25 to .41, and none of them was reliably different from zero (*p*s>.163). Thus, there was no clear linkage between long-term memory (either verbal or spatial) and performance in target-directed walking.

### Challenges for future research

In the present study, patients with epilepsy were compared against healthy controls who had no neurological problems. As a result, it is possible that the observed difference between the patients and controls in target-directed walking was caused by some general epilepsy-related effects (e.g., possible diffuse damage to the brain), rather than the absence of MTL structures. We suspect that this was not likely, given that the patients exhibited intact performance in all but one task; that is, it is unlikely that general effects would create such a specific behavioral dissociation between the patients and controls. Nevertheless, the possibility remains, and thus it is important to further investigate whether epilepsy itself could be a cause of path integration deficits.

It is also worth noting that the MTL is often associated with spatial information processing within allocentric (i.e., environment-centered), as opposed to egocentric (i.e., self-centered), frames of reference ([82], [88]; for a review, see [89]). This suggests that MTL-injured patients may be particularly impaired at tasks that involve allocentric spatial information. Examining the possible effects of spatial reference frames was beyond the scope of the present study, however, because most of the tasks employed in the present study allowed participants to process spatial information both allocentrically and egocentrically (e.g., in target-directed walking, participants were able to specify the target location relative to the surrounding hallway or themselves). Given the evidence that humans perform path integration by using both allocentric and egocentric strategies [90], it will be important in future work to clarify the extent to which differences in spatial reference frames or behavioral strategies affect the patients' performance in different path integration tasks.

As discussed above, the present results generally suggest that the modality of spatial information is not a decisive factor that determines the extent of MTL engagement during human path integration. However, it should be pointed out that a greater degree of multimodal integration of spatial information might have been required for target-directed walking than for experimenter-guided walking. In the target-directed walking task, the target location was specified visually, and this information had to be combined with vestibular and proprioceptive information acquired through walking. On the other hand, at least in principle, participants could have performed the experimenter-guided walking task on the basis of the vestibular and proprioceptive signals alone. Because vision of the environment in this task was provided before every trial, there is a very real possibility that participants engaged in some multimodal integration involving visual information even in the experimenter-guided walking task. Nevertheless, given the possible role of the MTL in integrating information from different modalities [91]–[93], it will be important to explore whether the potentially higher demand for multimodal integration in the target-directed walking task contributed to the observed dissociation between target-directed walking and experimenter-guided walking [94], [95].

Finally, as outlined earlier, MTL structures contain neurons that dynamically respond to an individual's location and heading in an environment (place, grid, and head-direction cells), suggesting that these structures play a role in path integration. However, neurons that exhibit properties similar to the grid cells are located outside the MTL as well [19], [23], in areas such as the posterior parietal and medial prefrontal cortices. This is consistent with evidence that these areas also perform neural computations underlying path integration [38], [39], [63], offering one possible explanation as to why deficits in target-directed walking after unilateral MTL injury are relatively small. Determining the extent to which intact performance could be carried out by these extra-MTL components of the brain's path integration network is an important challenge for future research [52], [96]–[98].

## Conclusions

This study demonstrated the MTL's participation in human path integration by showing that patients who underwent unilateral medial temporal lobectomy (in the right hemisphere, in particular) walked farther than age-matched healthy controls when attempting to walk to a previewed target (target-directed walking). However, the same patients performed normally when verbally estimating non-visually walked distances (experimenter-guided walking), even though these two tasks involved virtually the same kinds of sensory self-motion signals. These results suggest that the MTL's role in human path integration cannot be explained by strictly focusing on idiothetic signal processing, calling for a new viewpoint with which a variety of findings can be accommodated. One such viewpoint suggested by our results is that MTL structures play a role in producing the path integration benefits related to trajectory prediction–MTL-injured patients are unable to make full use of trajectory prediction signals to achieve the normal enhancement of path integration. This idea has several important strengths. First, it is consistent with data from multiple neuroscientific approaches (functional neuroimaging, behavioral testing in neurologically-intact and brain-injured humans, animal physiology, and computational movement neuroscience) [54], [65], [99]. Second, it provides parsimonious explanations for several apparent discrepancies in past work [47]–[51]. Third, our work also illuminates the conditions under which the MTL is likely engaged during path integration: specifically, tasks that involve physical movement toward salient targets (such as our target-directed walking task) may be especially powerful for engaging MTL structures.

## Supporting Information

Text S1 **Details of unsigned (absolute) error and variable error analyses.**
(PDF)

Table S1 Mean unsigned (absolute) errors of the three participant groups and results of statistical analyses for each task.
(PDF)

Table S2 Mean variable errors of the three participant groups and results of statistical analyses for each task.
(PDF)

Table S3 Mean signed, unsigned (absolute), and variable errors of the three participant groups and results of statistical analyses for the whole-body rotation task based on untransformed data.
(PDF)
