# Supplementary material for: Medial Temporal Lobe Roles in Human Path Integration
Source: PLoS One. 2014 May 6;9(5):e96583. doi: 10.1371/journal.pone.0096583 (PMC4011851; doi:10.1371/journal.pone.0096583)
Supplement: Text S1 — Details of unsigned (absolute) error and variable error analyses. (PDF) [file pone.0096583.s001.pdf]

Text S1. Details of unsigned (absolute) error and variable error analyses

In addition to signed errors, we computed unsigned (absolute) errors and analyzed them in the same manner as signed errors. (Details of the signed error analyses are shown in the main article.) The unsigned errors provide a measure of the overall amount of error without regard to the sign of error. We conducted these analyses because positive and negative errors can cancel out each other in signed error analyses, and therefore they could underestimate the total amount of error. Furthermore, we also measured within-subject variable errors, which provide a measure of response precision. To do this, we fit a straight line through the raw responses, plotted as a function of the physically correct values; we then calculated the standard error of estimate as a measure of overall response precision. These fits were generally excellent, with the average R^2 values ranging from .78 (time estimation) to .94 (target-directed walking). For the triangle completion, imagined walking, and third-person time-to-contact tasks, a smaller range of stimulus values was available, so we assessed response precision by calculating within-subject standard deviations across repetitions and then averaging these values.

Mean unsigned and variable errors for each group are presented in Tables S1 and S2, respectively, along with their standard errors and results of statistical tests for the main effect of group in each task. We also computed correlations between each type of error and the time of testing relative to surgery to examine possible effects of this variable on participants' performance. However, these correlations were small and none of them was reliably different from zero ($-.25 < r$s $< .22$, ps $> .255$ for unsigned errors; $-.34 < r$s $< .25$, ps $> .117$ for variable errors).
