# Supplementary material for: Medial Temporal Lobe Roles in Human Path Integration
Source: PLoS One. 2014 May 6;9(5):e96583. doi: 10.1371/journal.pone.0096583 (PMC4011851; doi:10.1371/journal.pone.0096583)
Supplement: Table S1 — Mean unsigned (absolute) errors of the three participant groups and results of statistical analyses for each task. (PDF) [file pone.0096583.s002.pdf]

Table S1. Mean unsigned (absolute) errors of the three participant groups and results of statistical analyses for each task

Task	F, p, and η^2 statistics[b]	Group means (and their standard errors)[a]		
		CONT[c]	LTLR[c]	RTLR[c]
Target-directed walking	$F_{(2, 32)} = .16$, $p = .854$, $\eta^2 = .01$	14.08 (1.54)	14.62 (1.77)	16.00 (3.86)
Experimenter-guided walking	$F_{(2, 32)} = .45$, $p = .640$, $\eta^2 = .03$	33.42 (2.73)	35.02 (3.49)	29.82 (5.41)
Verbal distance estimation	$F_{(2, 32)} = .45$, $p = .644$, $\eta^2 = .03$	24.76 (3.53)	28.49 (7.38)	20.98 (3.86)
Delayed distance matching	$F_{(2, 31)} = .42$, $p = .658$, $\eta^2 = .03$	12.03 (1.45)	12.83 (2.16)	14.32 (1.37)
Triangle completion[d]				
Response turn	$F_{(2, 32)} = .23$, $p = .796$, $\eta^2 = .01$	26.55 (1.06)	25.31 (1.59)	25.88 (1.21)
Response leg	$F_{(2, 32)} = 1.88$, $p = .169$, $\eta^2 = .11$	32.66 (2.14)	38.71 (3.12)	39.69 (2.87)
Whole-body rotation[e]	$F_{(2, 30)} = .08$, $p = .926$, $\eta^2 = .01$	27.70 (4.07)	24.31 (3.14)	28.39 (3.56)
Imagined walking[f]	$F_{(2, 32)} = 1.39$, $p = .263$, $\eta^2 = .08$	18.97 (3.63)	34.83 (9.08)	36.69 (10.96)
Blind pulling	$F_{(2, 32)} = .20$, $p = .821$, $\eta^2 = .01$	25.97 (2.13)	31.88 (10.21)	30.09 (3.86)
Third-person time-to-contact	$F_{(2, 32)} = .63$, $p = .541$, $\eta^2 = .04$	37.73 (5.27)	39.45 (11.18)	51.52 (9.23)
Time estimation	$F_{(2, 31)} = .56$, $p = .579$, $\eta^2 = .03$	41.11 (7.10)	59.14 (15.06)	27.99 (6.66)

[a] Except as noted, group means are expressed as a percentage of the correct response values.
[b] Statistics associated with the test of the main effect of group in each task. Degrees of freedom are not uniform across the tasks because some participants were not tested in all of the tasks. For details, see the results section of the main article.
[c] CONT = age-matched healthy control; LTLR = left temporal lobe resection; RTLR = right temporal lobe resection.
[d] See Table 2 in the main article for the stopping point error. Signed and unsigned stopping point errors were identical by definition, and thus they were not included here.
[e] The data reported in this table were corrected for possible errors in response execution by following the procedure described in the results section of the main article. Uncorrected data are shown in Table S3.
[f] Differences between mean imagined walking time and mean real walking time expressed as a percentage of the mean real walking time. For details, see the results section of the main article.
