# Supplementary material for: Medial Temporal Lobe Roles in Human Path Integration
Source: PLoS One. 2014 May 6;9(5):e96583. doi: 10.1371/journal.pone.0096583 (PMC4011851; doi:10.1371/journal.pone.0096583)
Supplement: Table S2 — Mean variable errors of the three participant groups and results of statistical analyses for each task. (PDF) [file pone.0096583.s003.pdf]

Table S2. Mean variable errors of the three participant groups and results of statistical analyses for each task

		Group means (and their standard errors)[a]		
**Task**	***F*, *p*, and η^2 statistics[b]**	**CONT[c]**	**LTLR[c]**	**RTLR[c]**
Target-directed walking	$F_{(2, 32)} = 1.62$, $p = .214$, $\eta^2 = .09$	.31 (.02)	.39 (.03)	.41 (.06)
Experimenter-guided walking	$F_{(2, 32)} = 2.14$, $p = .134$, $\eta^2 = .12$	.39 (.07)	.65 (.12)	.55 (.06)
Verbal distance estimation	$F_{(2, 32)} =1.17$, $p = .324$, $\eta^2 = .07$	.38 (.07)	.50 (.09)	.35 (.04)
Delayed distance matching	$F_{(2, 31)} = 1.32$, $p = .283$, $\eta^2 = .08$	.40 (.03)	.37 (.05)	.46 (.05)
Triangle completion				
Response turn	$F_{(2, 32)} = 2.53$, $p = .095$, $\eta^2 = .14$	10.78 (1.24)	7.97 (.65)	9.51 (.71)
Response leg	$F_{(2, 32)} = .90$, $p = .415$, $\eta^2 = .05$	14.77 (1.58)	13.91 (1.13)	16.80 (1.92)
Stopping point error	$F_{(2, 32)} = 1.10$, $p = .346$, $\eta^2 = .06$	.52 (.04)	.42 (.04)	.50 (.07)
Whole-body rotation[d]	$F_{(2, 30)} = .35$, $p = .709$, $\eta^2 = .02$	13.23 (1.89)	15.48 (2.18)	15.49 (2.57)
Imagined walking	$F_{(2, 32)} = .08$, $p = .928$, $\eta^2 = .01$	16.30 (1.81)	17.65 (3.58)	17.89 (3.26)
Blind pulling	$F_{(2, 32)} = .32$, $p = .729$, $\eta^2 = .02$	.57 (.08)	.59 (.15)	.46 (.07)
Third-person time-to-contact	$F_{(2, 32)} = 2.10$, $p = .140$, $\eta^2 = .12$	.80 (.12)	.98 (.20)	1.41 (.31)
Time estimation	$F_{(2, 31)} = .66$, $p = .522$, $\eta^2 = .04$	1.76 (.37)	2.18 (.64)	1.39 (.27)

[a] In most of the tasks, these errors were computed by fitting a straight line through the raw responses, plotted as a function of the physically correct values, and then calculating the standard error of estimate as a measure of overall response precision. For the triangle completion, imagined walking, and third-person time-to-contact tasks, mean within-subject standard deviations across repetitions were derived as variable errors. For details, see Text S1.

[b] Statistics associated with the test of the main effect of group in each task. Degrees of freedom are not uniform across the tasks because some participants were not tested in all of the tasks. For details, see the results section of the main article.

[c] CONT = age-matched healthy control; LTLR = left temporal lobe resection; RTLR = right temporal lobe resection.

[d] The data reported in this table were corrected for possible errors in response execution by following the procedure described in the results section of the main article. Uncorrected data are shown in Table S3.
