# Supplementary material for: Medial Temporal Lobe Roles in Human Path Integration
Source: PLoS One. 2014 May 6;9(5):e96583. doi: 10.1371/journal.pone.0096583 (PMC4011851; doi:10.1371/journal.pone.0096583)
Supplement: Table S3 — Mean signed, unsigned (absolute), and variable errors of the three participant groups and results of statistical analyses for the whole-body rotation task based on untransformed data. (PDF) [file pone.0096583.s004.pdf]

Table S3. Mean signed, unsigned (absolute), and variable errors of the three participant groups and results of statistical analyses for the whole-body rotation task based on untransformed data

		Group means (and their standard errors)[a]		
**Error type**	***F*, *p*, and η^2 statistics**[b]	**CONT**[c]	**LTLR**[c]	**RTLR**[c]
Signed	$F_{(2, 30)} = .29, p = .752, \eta^2 = .02$	8.85 (6.56)	32.67 (25.58)	26.46 (32.68)
Unsigned	$F_{(2, 30)} = .34, p = .715, \eta^2 = .02$	34.44 (9.95)	55.89 (23.10)	57.59 (30.23)
Variable	$F_{(2, 30)} = .23, p = .798, \eta^2 = .01$	21.33 (7.99)	19.05 (6.05)	26.82 (99.64)[d]

[a] Mean signed and unsigned errors are expressed as a percentage of the correct response values. Variable errors were computed by fitting a straight line through the raw responses, plotted as a function of the physically correct values, and then calculating the standard error of estimate as a measure of overall response precision.
[b] Statistics associated with the test of the main effect of group in each type of error.
[c] CONT = age-matched healthy control; LTLR = left temporal lobe resection; RTLR = right temporal lobe resection.
[d] There was an outlier in the RTLR group: one of the participants who exhibited possible response execution errors had a variable error of 113.07; the next highest variable error in this group was 27.01. When this participant was omitted, the group mean was 16.04 with a standard error of 22.82.
